# HLA-DQA1*05 correlates with increased risk of anti-drug antibody development and reduced response to infliximab in Chinese patients with Crohn’s disease

**DOI:** 10.1093/gastro/goae074

**Published:** 2024-07-24

**Authors:** Wei Wang, Qi Zhang, Junzhang Zhao, Tao Liu, Jiayin Yao, Xiang Peng, Min Zhi, Min Zhang

**Affiliations:** Department of Gastroenterology, The Sixth Affiliated Hospital, Sun Yat-Sen University, Guangzhou, Guangdong 510655, P. R. China; Biomedical Innovation Center, The Sixth Affiliated Hospital, Sun Yat-Sen University, Guangzhou, Guangdong 510655, P. R. China; Department of Gastroenterology, The Sixth Affiliated Hospital, Sun Yat-Sen University, Guangzhou, Guangdong 510655, P. R. China; Biomedical Innovation Center, The Sixth Affiliated Hospital, Sun Yat-Sen University, Guangzhou, Guangdong 510655, P. R. China; Department of Gastroenterology, The Sixth Affiliated Hospital, Sun Yat-Sen University, Guangzhou, Guangdong 510655, P. R. China; Biomedical Innovation Center, The Sixth Affiliated Hospital, Sun Yat-Sen University, Guangzhou, Guangdong 510655, P. R. China; Department of Gastroenterology, The Sixth Affiliated Hospital, Sun Yat-Sen University, Guangzhou, Guangdong 510655, P. R. China; Biomedical Innovation Center, The Sixth Affiliated Hospital, Sun Yat-Sen University, Guangzhou, Guangdong 510655, P. R. China; Department of Gastroenterology, The Sixth Affiliated Hospital, Sun Yat-Sen University, Guangzhou, Guangdong 510655, P. R. China; Biomedical Innovation Center, The Sixth Affiliated Hospital, Sun Yat-Sen University, Guangzhou, Guangdong 510655, P. R. China; Department of Gastroenterology, The Sixth Affiliated Hospital, Sun Yat-Sen University, Guangzhou, Guangdong 510655, P. R. China; Biomedical Innovation Center, The Sixth Affiliated Hospital, Sun Yat-Sen University, Guangzhou, Guangdong 510655, P. R. China; Department of Gastroenterology, The Sixth Affiliated Hospital, Sun Yat-Sen University, Guangzhou, Guangdong 510655, P. R. China; Biomedical Innovation Center, The Sixth Affiliated Hospital, Sun Yat-Sen University, Guangzhou, Guangdong 510655, P. R. China; Department of Gastroenterology, The Sixth Affiliated Hospital, Sun Yat-Sen University, Guangzhou, Guangdong 510655, P. R. China; Biomedical Innovation Center, The Sixth Affiliated Hospital, Sun Yat-Sen University, Guangzhou, Guangdong 510655, P. R. China

**Keywords:** Crohn’s disease, infliximab, HLA-DQA1*05, anti-drug antibodies

## Abstract

**Background:**

The efficacy of anti-TNF therapy in Crohn’s disease (CD), such as infliximab, is often compromised by the development of anti-drug antibodies (ADAs). The genetic variation HLA-DQA1*05 has been linked to the immunogenicity of biologics, influencing ADA formation. This study investigates the correlation between HLA-DQA1*05 and ADA formation in CD patients treated with infliximab in a Chinese Han population and assesses clinical outcomes.

**Methods:**

In this retrospective cohort study, 345 infliximab-exposed CD patients were genotyped for HLADQ A1*05A>G (rs2097432). We evaluated the risk of ADA development, loss of infliximab response, adverse events, and treatment discontinuation among variant and wild-type allele individuals.

**Results:**

A higher percentage of patients with ADAs formation was observed in HLA-DQA1*05 G variant carriers compared with HLA-DQA1*05 wild-type carriers (58.5% vs 42.9%, *P *=* *0.004). HLA-DQA1*05 carriage significantly increased the risk of ADAs development (adjusted hazard ratio = 1.65, 95% CI 1.18–2.30, *P *=* *0.003) and was associated with a greater likelihood of infliximab response loss (adjusted HR = 2.55, 95% CI 1.78–3.68, *P *<* *0.0001) and treatment discontinuation (adjusted HR = 2.21, 95% CI 1.59–3.06, *P *<* *0.0001). Interestingly, combined therapy with immunomodulators increased the risk of response loss in HLA-DQA1*05 variant carriers.

**Conclusions:**

HLA-DQA1*05 significantly predicts ADAs formation and impacts treatment outcomes in infliximab-treated CD patients. Pre-treatment screening for this genetic factor could therefore be instrumental in personalizing anti-TNF therapy strategies for these patients.

## Introduction

Identification of patients at higher risk of developing anti-drug antibodies (ADAs) is crucial for successful treatment with anti-tumor necrosis factor (anti-TNF) agents. Numerous factors have been linked to the failure of anti-TNF treatment, including patient-, disease-, and drug-related factors [1]; however, their relative contributions and interactions on drug concentrations and ADA formation remain incompletely understood. While the majority of existing studies have primarily focused on the association between the development of ADAs and clinical outcomes of infliximab (IFX), they have not explored the underlying causes of antibody formation [2–5]. Therefore, early identification of patients at risk of developing ADAs to anti-TNF agents may be crucial in mitigating the development of these antibodies and improving therapeutic strategies for patients.

IFX with immunomodulators or adalimumab may still be preferred as first-line therapy for induction of clinical remission in patients with moderate-to-severe Crohn’s disease (CD), despite the availability of individualized choices in biologic treatment [6, 7]. However, one-third of patients with CD fail to respond to anti-TNFα during the induction and maintenance period [8–10]. The immunogenicity of the biological drug IFX is an immediate drawback, as it can lead to increased formation of ADAs. These ADAs are associated with worse clinical outcomes and increased the clearance of drug, resulting in low-serum concentrations of IFX [2, 3, 11]. Therefore, optimized therapeutic strategies such as proactive therapeutic drug monitoring [12, 13], intensified dosages, shorter infusion intervals, or additional therapy with immunomodulators based on trough IFX concentration and ADAs have been implemented to improve the clinical outcome of IFX treatment [14–18]. However, these treatments solely address the potential adverse effects on clinical outcomes subsequent to ADA formation and do not delve into the underlying causes of ADA development.

Studies investigating the polymorphism of FCGR3A [19, 20], CD96 [21], and HLA-DRB1 [22] aim to shed light on the cellular and molecular mechanisms underpinning immunogenicity to biologics. However, these studies often have limitations due to small sample sizes and study designs. Previous studies have reported genetic locus HLA-DQA1*05 is robustly associated with immunogenicity to anti-TNF therapies in populations of European descent with inflammatory bowel disease [23, 24]. One study also demonstrated an association between ADA formation and genotype-dependent differences in a small sample size of Chinese CD populations receiving anti-TNFα therapy; however, no further analysis was conducted to investigate the significance of HLA-DQA1*05 carriage and clinical outcomes [20]. Here, we analyzed a large cohort of Han ethnic Chinese patients with CD who received IFX treatment and we hypothesized that genetic marker HLA-DQA1*05 also was associated with ADAs formation and loss of response to IFX. Overall, the primary objective of this study is to gain insights into the correlation between this genetic factor and the development of ADAs, as well as its impact on clinical outcomes in patients with CD undergoing IFX treatment.

## Methods

### Study subjects and enrollment procedures

**Figure 1.**
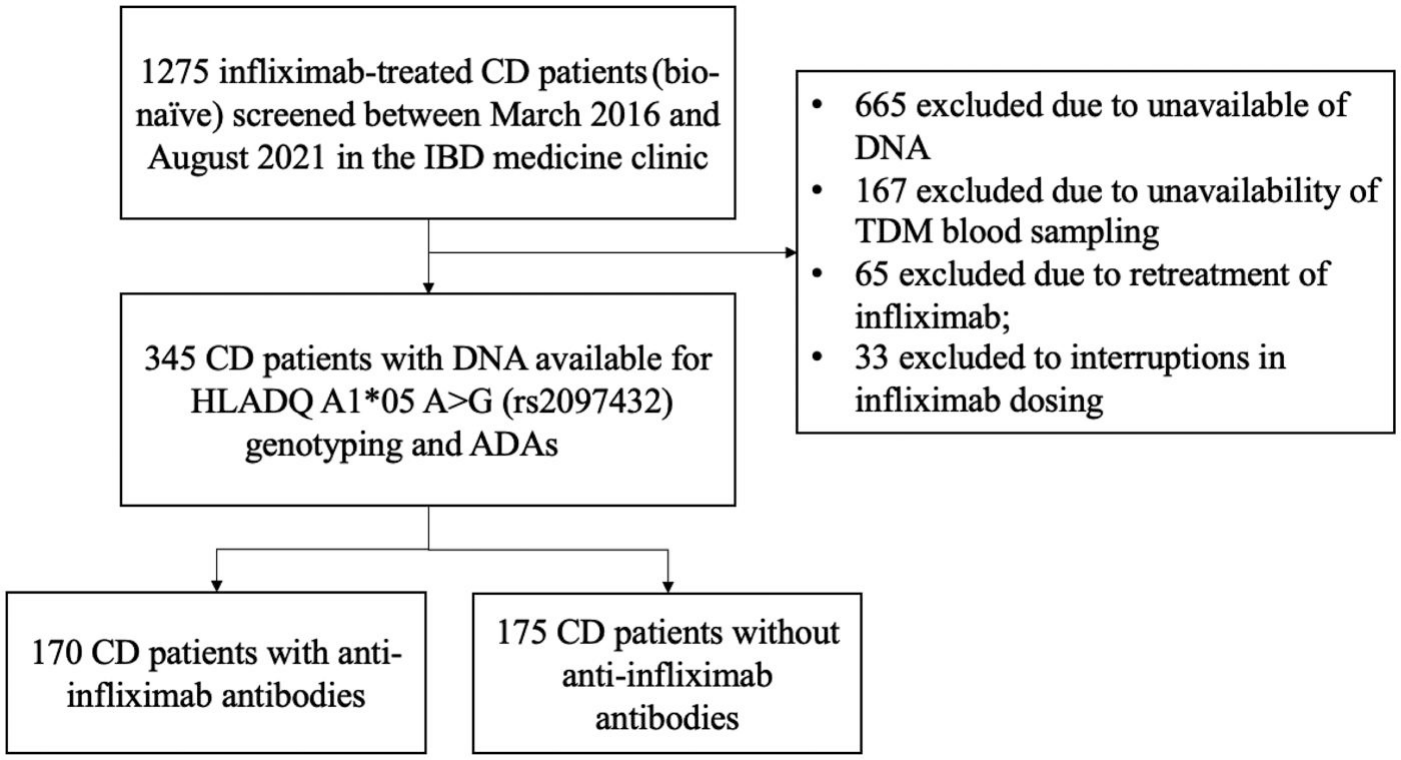
Flowchart of this study. DNA = deoxyribonucleic acid, IBD = inflammatory bowel disease, TDM = therapeutic drug monitoring.

This single-center retrospective cohort study was conducted with the approval of the Ethics Board of the Sixth Affiliated Hospital, Sun Yat-Sen University (Guangzhou, Guangdong, P. R. China). We studied 1,275 adults with bio-naïve CD receiving IFX treatment (5 mg/kg, originator, Remicade, Cilag AG, Schaffhausen, Switzerland) between March 2016 and August 2021. Eligible participants were adults (aged 18 years or older) with active disease, either beginning or maintaining immunomodulator therapy with IFX. We excluded patients who lacked therapeutic drug monitoring data or those undergoing re-treatment with IFX (Figure 1). Ultimately, 345 individuals met these criteria and were included in our study. The study had a minimum follow-up of 6 months, concluding on 1 March 2022. This allowed us to conduct a comprehensive analysis over a significant duration. Assessments were strategically conducted at baseline, post-induction (Weeks 12–14), Week 54 following treatment initiation, and in cases of treatment failure. We collected patient demographics data and medication records from the start of IFX therapy until the end of the study period (1 March 2022).

### Study objectives

The study defined drug persistence as the duration from initiation of IFX therapy to either treatment failure or end of follow-up. Clinical failure was identified by a CD active index score of 150 or higher with recurrence of symptoms, and required a switch to an alternative treatment regimen for re-induction of remission after Week 14 of IFX, and also in cases where adverse events led to discontinuation of treatment [25]. The primary endpoint was to evaluate the risk of forming ADAs against IFX. Secondary outcomes included evaluating the risk associated with IFX therapy, such as loss of response, discontinuation of treatment, and occurrence of drug-related adverse events [26, 27].

### ADA detection

Plasma was sampled and utilized for the detection of trough drug concentration during each visit prior to IFX injection following the initiation of the first dose of IFX. Serum IFX drug and ADA concentration were consistently analyzed by a drug-sensitive, commercially available enzyme-link immunosorbent assay to IFX (Immundiagnostik, Bensheim, Germany) at each visit. Immunogenicity was defined as ADA concentrations of 10 AU/mL or higher at one or more time points in the absence of detectable serum IFX.

### Genotypic analysis of HLA-DQA1*05

DNA was extracted from whole blood samples from all participants using a commercially used DNA extraction kit (BIOG Blood DNA Isolate Kit, BAIDAI, Changzhou, Jiangsu, P. R. China). The amplification primer (forward: 5′-CAGTTTAGTCTGCCAGAGACAAATG-3′, reverse: 5′-AAGCCCAGTCTCTTTCAGATGTT-3′) was designed based on rs2097432 (chromosome 6) sequence and specificity was verified. The polymerase chain reaction (PCR) conditions and parameters were performed as follows: preliminary denaturation was conducted at 95°C for 5 min, followed by 35 cycles of denaturation at 95°C with annealing at 58°C for 30 s and extension at 72°C for 30 s, and a final extension at 72°C for 5 min. PCR products were used for HLA-DQA1*05 typing based on Sanger sequencing high resolution.

### Statistical analyses

Statistical analysis was performed using the SPSS 23.0 statistical package (IBM SPSS, Chicago, IL, USA) and GraphPad Prism version 8. Hardy–Weinberg equilibrium was tested using a chi-squared goodness-of-fit test to assess HLA-DQA1*05 allele frequency. Statistical differences between HLA-DQA1*05 wild-type and variant carries were analyzed by Student’s *t* test for continuous variables and Fisher’s exact test. Patients who did not develop ADAs during the study were censored at the point of last observation. Kaplan–Meier method was used to estimate rates of immunogenicity. Cox proportional hazards regression was used to estimate clinical outcomes and genetic association, adjusting for age, sex, weight, and immunomodulator use.

## Results

### Baseline characteristics of enrolled subjects

The baseline characteristics of the study participants are summarized in Table 1. A total of 345 patients with CD were included in the study. The age and median disease duration were similar between the group of patients who developed anti-IFX antibodies and the group who did not. Additionally, a statistically significant association was observed between lower body weight and the development of anti-IFX antibodies in patients receiving IFX treatment (*P *<* *0.001).

**Table 1.** Demographic characteristics of enrolled patients (*n *=* *345)

Variables	Anti-infliximab antibodies (*n *=* *170)	No anti-infliximab antibodies (*n *=* *175)	*P*-value
Age, years, median (IQR)	26.32 (20–30)	26.43 (21–31)	NS
Male, *n* (%)	121 (71.2%)	139 (79.4%)	NS
Weight, kg, mean±SD	51.2 ± 9.6	55.2 ± 9.3	<0.001
Median disease duration, years, mean ± SD	2.5 ± 2.9	2.8 ± 4.3	NS
Crohn’s disease, *n*			
L1	14 (L4, 4)	6 (L4, 1)	
L2	6 (L4, 1)	15 (L4, 1)	
L3	150 (L4, 16)	154 (L4, 15)	
Disease behavior, *n* (%)			
B1	105 (61.8%)	111 (63.4%)	
B2	23 (13.5%)	25 (14.3%)	
B3	42 (24.7%)	39 (22.3%)	
P	119 (70.0%)	107 (61.1%)	
CDAI, mean±SD	210.51 ± 77.89	210.17 ± 73.96	0.820
Current smoking	0 (0)	0 (0)	NS
Surgery	0 (0)	0 (0)	NS
Combination therapy with immunomodulators	78 (45.9%)	70 (40.0%)	NS
Immunomodulators exposure before infliximab	64 (37.6%)	67 (38.3%)	NS
Loss of response	90 (52.9%)	42 (24.0%)	<0.001
Discontinuation	102 (60.0%)	58 (33.1%)	<0.001

CDAI = Crohn’s disease active index, IQR = interquartile range, SSD = standard deviation, NS = no significant, L1 = ileal, L2 = colonic, L3 = ileocolonic location of disease, L4 = upper gastrointestinal, B1 = inflammatory disease, B2 = stricturing disease, B3 = penetrating disease, P = perianal disease.

### Frequency of HLA-DQA1*05 and clinical outcomes

**Figure 2.**
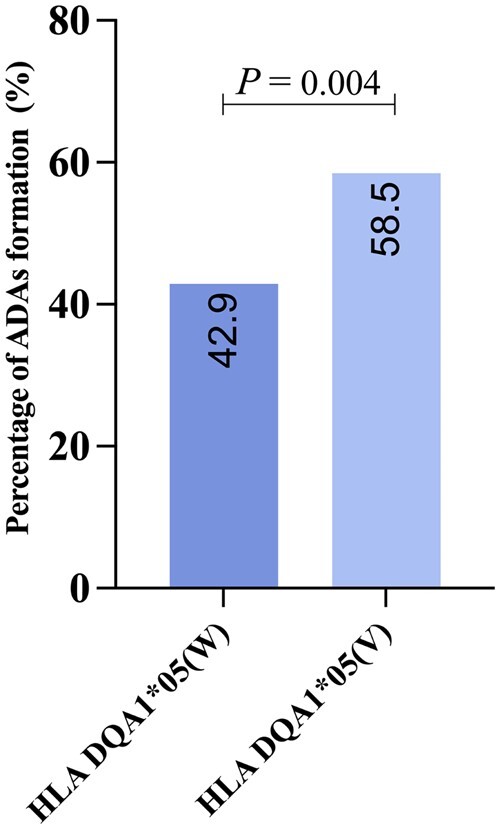
Genotype was associated with HLA-DA1*05 rs209743 carriage. A higher percentage of patients with ADAs formation in HLA-DQA1*05 G variant carriers compared with HLA-DQA1*05 wild-type carriers (58.5% vs 42.9%, *P *=* *0.004). W = wild-type HLA-DA1*05, V = variant HLA-DA1*05.

The HLA-DQA1*05 A > G (rs2097432) locus demonstrated Hardy–Weinberg equilibrium within our study population, indicating no significant deviation from expected genotype frequencies. The median follow-up for the entire study population was 81 weeks [interquartile range (IQR), 47–125 weeks]. We observed that IFX-related adverse events occurred in 17 of 345 patients (4.9%), including immediate infusion reaction (*n *=* *1), delayed infusion reaction (*n *=* *2), psoriasiform rash (*n *=* *1), anaphylactoid (*n *=* *8), infection (*n *=* *4), and tumor (*n *=* *1). However, there was no significant difference in the incidence of adverse events between HLA-DQA1*05 G (*n *=* *11) and wild-type HLA-DQA1*05 carriers (*n *=* *6; 5.4% vs 4.2%, *P *=* *0.419). Furthermore, patients who developed ADAs to IFX had a significantly higher incidence of loss of response compared with those without ADAs (52.9% vs 24.0%, *P *<* *0.001) at the end of the study, resulting in a higher discontinuation rate of IFX therapy in ADAs compared with non-ADAs groups in CD (60.0% vs 33.1%, *P *<* *0.001). We observed a higher percentage of patients with ADAs formation in HLA-DQA1*05 G variant carriers compared with HLA-DQA1*05 wild-type carriers (58.5% vs 42.9%, *P *=* *0.004; Figure 2), suggesting a potential association between carriage of the HLA-DQA1*05 allele and ADA formation.

### HLA-DQA1*05 carriage was associated with the outcomes of IFX therapy

**Figure 3.**
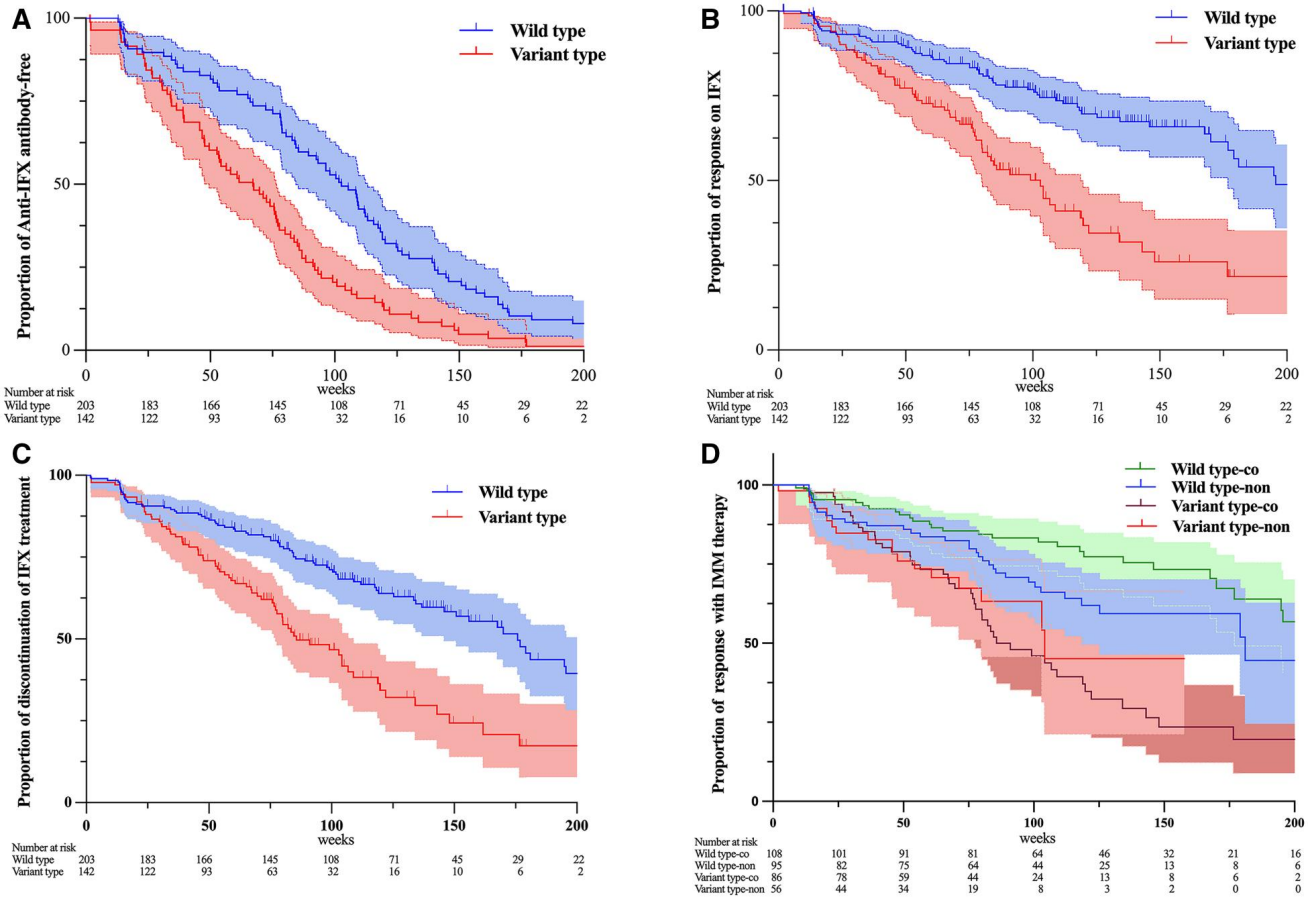
Adjusted survival curves of anti-infliximab antibody formation in HLA-DQA1*05 carriers. (A) Proportion of anti-IFX antibody-free in subjects with HLA-DQA1*05 carriers and non-carriers. (B) Proportion of response on IFX in subjects with HLA-DQA1*05 carriers and non-carriers. (C) Proportion of discontinuation of IFX treatment in subjects with HLA-DQA1*05 carriers and non-carriers. (D) Proportion of response with IMM therapy. These data were adjusted with age, gender, weight, and combined immunomodulators (azathioprine or methotrexate). 95% confidence intervals are represented by different colors as presented in photographs. Type-co, co-immunomodulators; type-non, no co-immunomodulators.

Patients carrying the HLA-DQA1*05 G allele who developed ADAs had a median time of 28 weeks (IQR, 15.0–51.0) for anti-IFX antibody formation and 53 weeks (IQR, 28.5–77.5) for loss of response, compared with 31 weeks (IQR, 16.0–67.0) and 80 weeks (IQR, 36.5–115.0), respectively, in wild-type individuals. The carriage of HLA-DQA1*05 G allele significantly increased the risk of developing anti-IFX antibody, even adjusting for gender, age, weight, and concurrent use of immunomodulators (azathioprine or methotrexate) [adjusted hazard ratios (HR)=1.65, 95% CI 1.18–2.30, *P *=* *0.003; Figure 3A and Supplementary Table 1]. Furthermore, HLA-DQA1*05 G carriage was significantly associated with an elevated risk of loss response to IFX treatment (adjusted HR = 2.55, 95% CI 1.78–3.68, *P *<* *0.001; Figure 3B and Supplementary Table 2). Patients carrying HLA-DQA1*05 G allele exhibited a higher rate of discontinuation of IFX therapy (adjusted HR = 2.21, 95% CI 1.59–3.06, *P *<* *0.001; Figure 3C and Supplementary Table 3). HLA-DQA1*05 G carriage was not associated with an increased risk of adverse events (relative risk = 0.77, 95% CI 0.28–2.13, *P *=* *0.25). Combining immunomodulators with IFX significantly improved the remission rate at the end of the study in individuals carrying HLA-DQA1*05 wild-type carriers compared with those receiving no combined therapy or individuals carrying HLA-DQA1*05 G variant-type carriers, as evidenced by loss of response observation (HR = 0.38, 95% CI 0.35–0.92, *P *=* *0.024; Figure 3D). However, combination therapy with immunomodulators in HLA-DQA1*05 G variant-type carriers was associated with an increased risk of loss of response (HR = 1.99, 95% CI 1.29–3.11, *P *=* *0.021; Supplementary Table 4).

## Discussion

The development of ADAs to biological therapies is a significant concern for clinicians and patients alike. Unfortunately, there remains a dearth of direct methods and means to predict in advance which patients are at risk for ADAs formation and subsequent loss of response to IFX. Our study aimed to address this issue by investigating the association between HLA-DQA1*05 (rs2097432) carriage and the development of IFX-induced ADAs. Our findings indicated that CD subjects who carry with HLA-DQA1*05 variant had a higher prevalence of ADAs compared with those with HLA-DQA1*05 wild-type carriage. Furthermore, HLA-DQA1*05 carriers were more likely to develop ADAs against IFX and those carrying HLA-DQA1*05 G allele had a greater likelihood of experiencing loss of response and discontinuation of IFX therapy, even after adjusting for age, gender, weight, and co-immunomodulators. Moreover, we observed no correlation between HLA-DQA1*05 carriage and drug-related adverse events. Furthermore, our study demonstrated that combined therapy with immunomodulators could enhance the clinical outcome in HLA-DQA1*05 wild-type carriers, but not those with variant carriage.

HLA-DQA1*05 carriage has been associated with various diseases, including celiac disease, type I diabetes, and protection against rheumatoid arthritis [28–30], suggesting that allelic variation in the *HLA-DQA1* gene may contribute to aberrant adaptive immune responses. The HLA class II gene *HLA-DQA1* is expressed in antigen-presenting cells and encodes the α-chain of the HLA-DQ heterodimer, which forms part of the antigen-binding site where epitopes are presented to T-helper cells. Immunogenicity prediction is based on well-defined interactions between the amino acids that make up a protein sequence and an individual’s HLA molecules, aside from the influence of T cell receptor affinity; the greater the stability of a given peptide within the binding groove of a particular HLA class II molecule, the more likely it is that this peptide will elicit a T cell response [31, 32].

In this study, we identified a significant association between HLA-DQA1*05 carriage and the development of ADAs formation as well as loss of response to IFX in patients with CD. These findings are consistent with previous studies conducted on European descent populations using genome-wide association or PCR-based detection methods [23, 24, 33]. Genetic research has demonstrated that HLA-DQA1*05 is not linked to an elevated risk of developing CD in individuals of European descent [34]. The percentage of patients carrying the HLA-DQA1*05 allele in our study (41%) was comparable to that reported in European descent populations (38%–39%) [35]. Several studies have shown that variations in the highly polymorphic HLA gene are linked to hypersensitivity reactions to various drugs [36–38], indicating that HLA-DQA1*05 may serve as a valuable biomarker for predicting immunogenicity risk. However, the presence of HLA-DQA1*05 variant carriage alone did not consistently result in ADA formation, suggesting that additional factors may contribute to immunogenicity. Studies investigating the polymorphism of FCGR3A [19, 20], CD96 [21], and HLA-DRB1 [22], as well as patients harboring two variants (rs2097432 in HLA-DQA1*05 and rs396991 in FCGR3), exhibited a higher proportion of producing ADAs [20]. However, these studies were conducted with limited sample size and did not extensively explore the correlation with clinical outcomes, therefore, further investigations should be undertaken to examine combined loci for predicting ADAS. Additionally, a previous study demonstrated that ADA production was correlated with the utilization of antibiotics within the 3-year period preceding the initiation of anti-TNF treatment [39]. In this study, it was challenged to adjust for the impact of antibiotics on antibody development based on the type and severity of the infection, germ-free mice exposure to IFX in a mouse model did not exhibit detectable antibodies, suggesting that antibiotic-induced dysbiosis was associated with antibody development. However, dysbiosis of the microbiota is prevalent in IBD [40–42], thus further investigation is needed to elucidate the influence of antibiotics on antibody development. In this study, we showed that concomitant administration of immunomodulators was associated with a decreased risk of loss of response to IFX in patients carrying HLA-DQA1*05 wild-type allele, whereas no such benefit was observed in those carrying the HLA-DQA1*05 variant allele. Combination therapy with thiopurine or methotrexate was found to mitigate the development of ADAs for both IFX and adalimumab, thereby enhancing drug persistence or retention in patients with CD [43–45]. However, our study revealed that combination therapy of IFX and immunomodulators in HLA-DQA1*05 variant carriers was associated with an increased risk of discontinuation of IFX treatment. The study showed that HLA-DQA1*05 variant carriers were associated with lower concentration of IFX during maintenance therapy [46], therefore, further investigation is warranted to explore the therapeutic efficacy of combined immunomodulators in individuals with CD who carry the HLA-DQA1*05 variant allele.

The retrospective nature of the analysis is a potential drawback. A selection bias might have occurred from the analysis of only patients with available ADA levels. In this study, we employed a free ADA enzyme-linked immunosorbent assay detection kit to detect IFX ADAs, however, it is important to acknowledge the limitations of this kit as it solely enables the detection of free antibodies rather than those that are bound to IFX. Therefore, it may not detect all ADAs present in samples, and it cannot distinguish between transient and sustained IFX ADAs. Previous studies demonstrated that IBD patients with both high IFX trough levels and ADAs also have worse treatment outcomes in mucosal healing during maintenance [2, 3]. Van Stappen *et al.* [47] also concluded that a drug-tolerant assay is not superior to a drug-sensitive assay for the management of patients with CD on maintenance remission. Secondly, we defined loss of response and primary non-response using CD active index score rather than endoscopic indices as our outcomes of interest. In future studies, it would be valuable to investigate the relationship between HLA-DQA1*05 variant carriers and antibody formation using endoscopic indices (Crohn’s Disease Endoscopic Index of severity). Thirdly, we acknowledge the short duration of median follow-up, which may have underestimated the contribution of HLA-DQA1*05 to immunogenicity. One strength of our study is the large sample size and long-term follow-up. Our findings show that HLA-DQA1*05 carriage is associated with the antibody formation to IFX and loss of response and drug discontinuation. This suggests that HLA-DQA105 could serve as a useful biomarker for identifying patients at risk of developing immunogenicity to IFX.

## Conclusions

Our study highlights and validates the significance of HLA-DQA1*05 as a crucial factor in predicting the risk of IFX ADA formation and its association with loss of response of IFX and treatment discontinuation. To reduce the risk of immunogenicity and potential clinical treatment failure of IFX, pre-treatment testing for HLA-DQA1*05 may aid in customizing the selection of anti-TNF therapy for patients with CD.

## Supplementary Material

goae074_Supplementary_Data
