# Supplementary material for: HLA-DQA1*05 correlates with increased risk of anti-drug antibody development and reduced response to infliximab in Chinese patients with Crohn’s disease
Source: Gastroenterol Rep (Oxf). 2024 Jul 24;12:goae074. doi: 10.1093/gastro/goae074 (PMC11269678; doi:10.1093/gastro/goae074)
Supplement: goae074_Supplementary_Data [file goae074_supplementary_data.docx]

| **Supplementary Table 1**. Hazard ratios for infliximab anti-drug antibody formation. | | | |
| --- | --- | --- | --- |
| Variable | Hazard ratio | 95% confidence interval | *P*-value |
| Female | 1.52 | 0.99–2.30 | 0.052 |
| Age | 1.03 | 1.01–1.05 | 0.006 |
| Weight | 1.03 | 1.01–1.05 | <0.001 |
| HLA DQA1*05 (variant type) | 1.65 | 1.18–2.30 | 0.003 |
| Co-immunosuppression with azathioprine or methotrexate | 0.55 | 0.40–0.74 | $<$0.001 |

| **Supplementary Table 2.** Hazard ratios for loss of response on infliximab. | | | |
| --- | --- | --- | --- |
| Variable | Hazard ratio | 95% confidence interval | *P*-value |
| Female | 1.35 | 0.88–2.07 | 0.16 |
| Age | 1.02 | 0.99–1.04 | 0.13 |
| Weight | 0.98 | 0.96–1.00 | 0.09 |
| HLA DQA1*05 (variant type) | 2.55 | 1.78–3.68 | <0.001 |
| Co-immunosuppression with azathioprine or methotrexate | 0.82 | 0.58–1.18 | 0.28 |

| **Supplementary Table 3.** Hazard ratios for infliximab treatment discontinuation. | | | |
| --- | --- | --- | --- |
| Variable | Hazard ratio | 95% confidence interval | *P*-value |
| Female | 1.41 | 0.95–2.08 | 0.081 |
| Age | 1.02 | 0.99–1.04 | 0.09 |
| Weight | 0.99 | 0.99–1.01 | 0.19 |
| HLA DQA1*05 (variant type) | 2.21 | 1.59–3.06 | <0.001 |
| Co-immunosuppression with azathioprine or methotrexate | 0.77 | 0.56–1.07 | 0.11 |

| **Supplementary Table 4.** Hazard ratios for infliximab IMM co-therapy (loss of response). | | | |
| --- | --- | --- | --- |
| Variable | Hazard ratio | 95% confidence interval | *P*-value |
| Female | 1.34 | 0.87–2.05 | 0.18 |
| Age | 1.02 | 0.99–1.04 | 0.13 |
| Weight | 0.98 | 0.96–1.01 | 0.14 |
| HLA DQA1*05-IMM (variant type-co) | 1.99 | 1.29–3.11 | 0.002 |
| HLA DQA1*05-IMM (variant type-non) | 1.49 | 0.81–2.64 | 0.18 |
| HLA DQA1*05-IMM (wild type-co) | 0.57 | 0.35–0.92 | 0.02 |

IMM = immunomodulators.
